# Human Oral Keratinocytes Challenged by *Streptococcus sanguinis* and *Porphyromonas gingivalis* Differentially Affect the Chemotactic Activity of THP-1 Monocytes

**DOI:** 10.1155/2022/9112039

**Published:** 2022-04-26

**Authors:** Huajing Li, Chaminda Jayampath Seneviratne, Lijian Jin

**Affiliations:** ^1^Faculty of Dentistry, The University of Hong Kong, Hong Kong SAR, China; ^2^Department of Stomatology, Shenzhen People's Hospital (The Second Clinical Medical College, Jinan University; The First Affiliated Hospital, Southern University of Science and Technology), Shenzhen, Guangdong, China; ^3^National Dental Research Institute Singapore, National Dental Centre Singapore, Singapore; ^4^Oral Health ACP, Duke NUS Medical School, Singapore

## Abstract

Periodontal diseases are initiated by the shift from microbe-host symbiosis to dysbiosis, and the disrupted host response predominantly contributes to tissue destruction. This study investigated whether and to what extent human oral keratinocytes (HOKs) challenged by a periodontal commensal or pathogen could differentially affect the chemotactic activity of THP-1 monocytes. A selected periodontal commensal (*Streptococcus sanguinis* ATCC 10556) and a pathogen (*Porphyromonas gingivalis* ATCC 33277) were cultured and inoculated, respectively, into the lower chamber of Transwell® Permeable Supports with HOKs and incubated for 2 h or 18 h at 37°C under appropriate cell growth conditions. HOKs alone served as the control for the transwell migration assay. Well-stained THP-1 monocytes were seeded in the top chamber of the device, incubated for 2 h and then collected from the lower well for quantitation of the migrated fluorescence-labeled cells by the FACSCalibur™ flow cytometer. The statistical significance was determined using one-way ANOVA. The HOKs challenged by *S*. *sanguinis* attracted a significantly higher number of THP-1 cell migration as compared with the control after 2 h or 18 h interaction (*p* < 0.01). By contrast, *P*. *gingivalis*-treated HOKs exhibited a markedly reduced chemotactic effect on THP-1 cells (*p* < 0.01, 2 h; *p* < 0.05, 18 h). There was no significant difference in THP-1 cell migration among the groups with either *S*. *sanguinis* or *P*. *gingivalis* alone. The current findings on *P*. *gingivalis*-HOKs interactions with resultant paralysis of THP-1 cell chemotaxis provide further evidence that the keystone periodontopathogen *P*. *gingivalis* can evade innate defense and contribute to periodontal pathogenesis.

## 1. Introduction

Oral and gingival epithelia act as the frontline barriers of innate host defense and constantly interact with the external environment [1]. The intercellular junctions of epithelia consisting mostly of desmosomes are highly resistant to mechanical injury [2]. These stratified squamous epithelial cells interact closely and dynamically with supra- and subgingival plaque biofilms. Oral gingival epithelia have been previously thought to act as a physical defense only, while their active role as a biological barrier in the maintenance of tissue homeostasis has been highlighted [3]. Periodontal diseases are initiated by the shift of microbe-host symbiosis to dysbiosis, and notably, the disrupted immunoinflammatory response is predominantly involved in the destruction of periodontal tissues [4, 5]. On the other hand, the invasion of major periodontopathogens like *Porphyromonas gingivalis* into host cells could damage the tight junctions of epithelial cells [6] and the vascular endothelial barrier [7], and yet markedly enhance their pathogenicity. This is an important mechanism that these pathogens have intrinsically evolved to subvert and evade immune responses [8], thereby greatly contributing to the initiation and progression of periodontal diseases and common inflammatory comorbidities such as cardiovascular disease and Alzheimer's disease [7]. Therefore, further investigation into the interactive profiles of commensals and pathogens with host cells and the relevant implications is of great importance on a deep understanding of periodontal pathogenesis and developing novel strategies for precise oral healthcare in the near future.

Monocytes and macrophages play crucial roles in innate and acquired immune responses. Chemotaxis is a kind of cellular migration toward or away from defined niches of noxious stimuli via appropriate gradients of chemical concentration [9]. Conceivably, the activated monocytes and macrophages would be attracted or continue to attract other immune cells to the infection foci and quickly expand the inflammatory cascade reaction. It is a well-defined immunoinflammatory response in microbe-host cross-talk and interactions.

Oral epithelial cells and leukocytes exhibit different responses to the challenge of commensals and pathogens. It remains unclear whether the interactions of commensals or pathogens with oral epithelial cells could differentially induce the chemotactic effects on leukocytes like monocytes. The present study attempted to test the hypothesis that human oral keratinocytes (HOKs) challenged by a periodontal commensal (e.g., *Streptococcus sanguinis*) or a pathogen (e.g., *P*. *gingivalis*) could result in differential chemotactic effects on THP-1 monocytes. Herein, the null hypothesis was that there was no difference in the chemotactic effect on THP-1 monocytes resulting from the HOKs challenged by *S*. *sanguinis* or *P*. *gingivalis*.

## 2. Methods

### 2.1. The Apparatus of Transwell® Permeable Supports

The Transwell® Permeable Supports (Corning Incorporated, USA) as an established tool to conduct chemotactic assays *in vitro* were used in the experiment [10–12]. It consisted of upper and lower chambers, and the porous bottom of the insert provided independent access to both sides of cells, thereby allowing the membrane to serve as a good model to assess cell transport and chemotactic migration. Herein, the diameter of the transwell used in this study was 6.5 mm (for 24 well plates) with pore sizes of 8 *μ*m, allowing monocytes to migrate through as documented in previous studies [10, 12].

### 2.2. Culture of Bacteria

The commensal bacterium *Streptococcus sanguinis* (ATCC 10556) and key periodontopathogen *P*. *gingivalis* (ATCC 33277) were acquired from the archival collection at the Central Research Laboratories, Faculty of Dentistry, the University of Hong Kong. The bacteria were cultured and maintained as previously described [13, 14]. *S*. *sanguinis* was inoculated on a Columbia blood agar plate (Oxoid™, UK) containing 5% defibrinated horse blood (Hemostat, USA) two days prior to the experiment, and *P*. *gingivalis* was maintained on the same blood agar plate one week before the experiment, both in an anaerobic chamber at 37°C. The bacterial cells were then harvested after washing with sterile phosphate-buffered saline, and the suspensions were used for the subsequent assays.

### 2.3. Culture of Mammalian Cells

Primary HOKs were obtained commercially and subcultured in serum-free oral keratinocytes medium (OKM, ScienCell Research Laboratories, USA) with 1% of growth supplement and 1% of penicillin/streptomycin solution (ScienCell Research Laboratories, USA) following our established method [15]. Third passage HOKs cells which attached to the bottom surface were then cultured in 24-well transwell plates at a concentration of 5 × 10^3^ cells/cm^2^ in OKM without antibiotics and grown at 37°C in a humidified incubator with 5% CO_2_ until confluence.

THP-1 monocytes (ATCC® TIB-202™) were acquired from American Type Culture Collection (Manassas, USA), and the cells were seeded in ATCC-formulated RPMI-1640 Medium (ATCC, Manassas, USA) with 0.05 mM 2-mercaptoethanol and 10% fetal bovine serum (FBS) in 25T flasks with a density of 2 × 10^5^ cells/ml and subcultured when reached the density of 8 × 10^5^ cells/ml at 37°C in a humidified incubator of 5% CO_2_. The medium was changed every other day, and the RPMI-1640 medium without FBS was used on the day prior to the experiments. THP-1 cells were then cultured in 25T flask until about to confluence.

### 2.4. Staining of THP-1 Cells and Observation under Fluorescent Microscope

Upon confluence, the resuspended THP-1 cells were gently placed in 1 *μ*M of prewarmed CellTracker™ Probes working solution (Invitrogen Corp., USA). The cells were incubated for 30 min under appropriate growth conditions. The cells were then washed using prewarmed, nonserum-containing media and incubated by replacing the dye working solution with fresh, prewarmed media, and the cells were further incubated for 30 min at 37°C twice. Cellular staining was carefully checked after washing with fluorescence microscopy and a confocal laser scanning microscopy (CLSM) system (FLUOVIEW FV 1000, Olympus, Japan).

### 2.5. Bacteria-HOKs Interactions in the Transwell Apparatus

**Figure 1:**
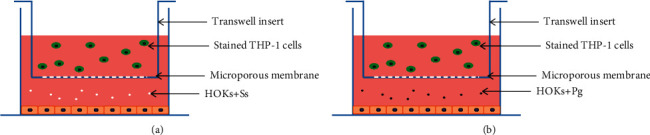
The models for chemotactic effect transwell apparatus. (a) HOKs challenged by commensal *S*. *sanguinis* (Ss); (b) HOKs challenged by periodontopathogen *P*. *gingivalis* (Pg).

*S. sanguinis* or *P. gingivalis* were inoculated into the lower chamber with HOKs with a multiplicity of infection 10 as suggested in [15] and incubated for 2 h or 18 h at 37°C in appropriate cell growth conditions. Successfully stained THP-1 cells were resuspended and seeded in the top chamber of the Transwell® Permeable Supports (Corning Incorporated) and then incubated for 2 h (Figure 1). *S*. *sanguinis*, *P*. *gingivalis*, and HOKs alone in the lower compartment served as the control groups, respectively.

### 2.6. Flow Cytometry

THP-1 cells were then collected from the lower well and assessed by running through a FACSCalibur™ flow cytometer (BD Biosciences, USA) for quantitation of the fluorescence-labeled cells that penetrated the membrane during incubation. Separately stained and unstained THP-1 cells were used for positive and negative controls, respectively.

### 2.7. Statistical Analysis

The results were presented as mean ± standard deviation, based on three independent assays. As the normality test and homogeneity of variances were justified to raw or transformed data, the statistical significance was determined using one-way ANOVA with SPSS Statistics for Windows, version 21.0 (IBM Corp. USA). A Bonferroni correction was made for intergroup comparison. Significant difference was defined with a *p* value <0.05.

## 3. Results

### 3.1. Staining of THP-1 Cells

**Figure 2:**
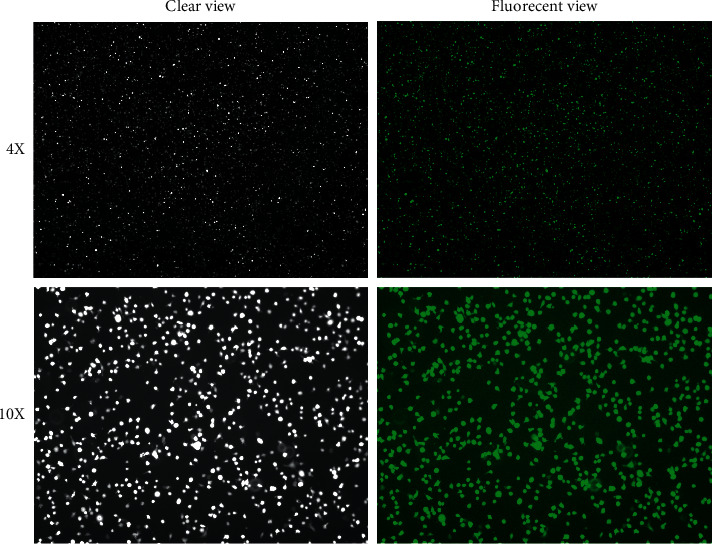
Clear and fluorescent views of cell tracker stained THP-1 cells ready for experiments with 4X or 10X magnification.

The successfully stained THP-1 cells are shown in Figure 2. Different layers of the suspension cells could still be observed under clear view or fluorescent view. The cells were used in the subsequent experiments.

### 3.2. Chemotactic Effect on THP-1 Cells at 2 h

**Figure 3:**
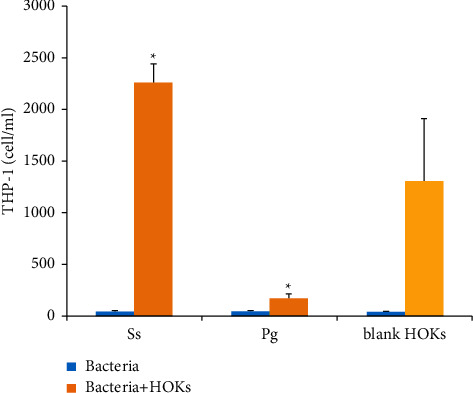
The number of THP-1 cells at 2 h attracted to the lower compartment of the transwell apparatus by bacteria alone, or the HOKs challenged by *S*. *sanguinis* (Ss) or *P*. *gingivalis* (Pg) for 2 h. Controls: blank (light blue) and HOK alone (yellow). The results are obtained from three independent assays. Significant difference from the control group, ^*∗*^*p* < 0.01.

As shown in Figure 3, the amount of stained THP-1 cells identified in the lower compartment of the transwell apparatus was significantly different among the groups. It was noteworthy that HOKs alone as the control could attract a notable number of THP-1 cells. The *S*. *sanguinis*-HOKs interactive group attracted a significantly higher number of THP-1 cells than that of the control (HOKs alone), while the *P*. *gingivalis*-challenged HOKs markedly reduced their basal chemotactic effect on THP-1 cells (*p* < 0.01). Moreover, the number of THP-1 cells in the *S*. *sanguinis*-HOKs group was over 10 times that of the *P*. *gingivalis*-HOKs group, with a significant intergroup difference (*p* < 0.01). Of note, either *S*. *sanguinis* or *P*. *gingivalis* alone could not effectively attract THP-1 cells, with reference to the blank control.

### 3.3. Chemotactic Effect on THP-1 cells at 18 h

**Figure 4:**
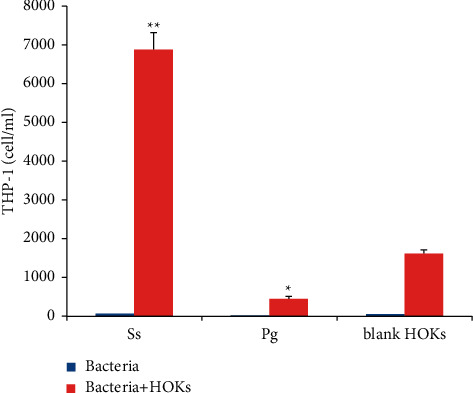
The number of THP-1 cells at 18 h attracted to the lower compartment of the transwell apparatus by bacteria alone, or the HOKs challenged by *S*. *sanguinis* (Ss) or *P*. *gingivalis* (Pg) for 18 h. Controls: blank (light blue) and HOK alone (light pink). The results are obtained from three independent assays. Significant difference from the control group, ^*∗*^*p* < 0.05 and ^*∗∗*^*p* < 0.01.

Significant difference remained to exist in THP-1 cell counts of the lower compartments among all groups after 18 h bacteria-HOKs interactions. Similarly, HOKs alone attracted a significant number of THP-1 cells. The *S*. *sanguinis*-HOKs interactive group markedly attracted a greater number of THP-1 cells with reference to the control (HOKs alone) (*p* < 0.01), whereas the migrated number of THP-1 cells in the *P*. *gingivalis-*HOKs interactive group was greatly less than the control (*p* < 0.05), being around 15 times as much cells detected in the former group than the latter one (*p* < 0.01). Furthermore, THP-1 cells could not be notably attracted by *S*. *sanguinis* or *P*. *gingivalis* alone (Figure 4).

## 4. Discussion

Periodontal diseases are multifactorial inflammatory conditions initiated by dysbiotic plaque biofilms and develop progressively through aberrant and dysregulated immunoinflammatory responses [16]. In fact, a vast majority of periodontal tissue destruction results from destructive host responses [17, 18]. Oral/gingival epithelia and other immune cells such as polymorphonuclear cells (PMNs), monocytes, and antigen-presenting cells (APCs) are important players in the response to microbial challenge. Chemotaxis is the magnificent step where all the immune cells get attracted to travel either toward or away from chemical stimuli to the locus of infection [19]. The transepithelial migration of immune cells could be one of the key mechanisms to control microbial challenges in the maintenance of periodontal homeostasis. The Transwell® Permeable Supports used in the present study have been shown to be a valid model to assess the chemotactic effect in immune cells [12, 20].

The present findings reject the null hypothesis and suggest that *S*. *sanguinis* as a commensal could well induce a moderate level of chemotaxis in THP-1 cells, while the keystone periodontopathogen, *P*. *gingivalis*, fails to attract immune cells for migration. Madianos and co-workers also showed that *P*. *gingivalis-*stimulated epithelial cells could not trigger effective migration of PMNs [21]. However, a previous study demonstrates that the supernatants from cultured mononuclear cells infected by different Gram-negative bacteria could trigger chemotaxis of monocytes [22], although the extent may be different depending on the differential species investigated. This observation suggests that the chemotactic effects may depend on the nature of the stimulating species. It could be postulated that the major periodontopathogen, *P*. *gingivalis*, may specifically develop such evasive strategy to manipulate innate host response. In fact, the bacteria-treated THP-1 cells could trigger the differentiation of monocytes to macrophages; therefore, the initial challenge of bacteria to THP-1 cells could lead to produce a combined pool of monocytes and macrophages when the infection occurs [23, 24]. This could to some extent mimic the i*n vivo* situation during the activation of monocytes.

Chemokine concentration is one of the important factors that may influence the migration of immunoinflammatory cells. Our recent work reveals that *P*. *gingivalis* could induce a high level of chemokine (e.g., IL-8) in HOKs, while the commensals may induce these chemotactic agents to a different extent depending on the species tested [15]. Although it seems surprising that *P*. *gingivalis* could not mount a significant level of chemotaxis on monocytes, it can be due to other underlying mechanisms, such as virulence factors like gingipains and/or negative feedback of chemical attractant concentration during the process of immunoinflammatory response [25]. Most of the proteolytic activity exhibited by *P*. *gingivalis* is due to Arg-gingipain (Rgp) and Lys-gingipain (Kgp) cysteine proteinases [26].

In addition, the pioneering study on microbial chemotaxis shows that bacteria like *E*. *coli* have chemotactic capacity toward various energy sources [27]. However, it is noteworthy that there are no strong chemotactic effects of bacteria *per se* observed in the present study, implying that the interactions of periodontal commensals (e.g., *S*. *sanguinis*) with nonmyeloid cells like HOKs are crucial for exerting effective chemotactic activities of myeloid cells in regulating immunoinflammatory responses to microbial challenge for maintaining tissue homeostasis. Further study is required to clarify these points.

Some limitations of this study need to be elaborated. Firstly, bacteria, especially for the anaerobic *P*. *gingivalis*, after incubation for an extended period of time (e.g., 18 h), become substantially less viable, which may to some extent affect the chemotactic effects observed. As such, this chemotaxis assay is unlikely to reflect the live bacteria-host interactions in a host system *in vivo* (e.g., 18 h). Secondly, in the present *in vitro* study, a single bacterial species-host cell interactive model is adopted as to eliminate the complexity of less controllable interference on the experiments. Further investigations on the interactions of multispecies biofilms with oral and gingival epithelial cells mimicking live bacteria-host crosstalk should be performed, yet the underlying mechanisms need to be further explored to enhance our understanding on the exact molecular pathways by which *P*. *gingivalis* evades innate host defense and crucially contributes to periodontal pathogenesis. The present study would contribute to developing novel strategies and approaches to tackling *P*. *gingivalis* for effective oral and periodontal healthcare.

In conclusion, this study shows that the interactions of HOKs with *P*. *gingivalis* exhibit significantly less chemotactic effects on THP-1 monocytes, with reference to those challenged by the periodontal commensal *S*. *sanguinis*. These findings demonstrate that *P*. *gingivalis* as a keystone periodontopathogen is able to evolve and develop intrinsic mechanisms to evade innate host defence and crucially account for the initiation and progression of periodontal diseases.

## Data Availability

The datasets used and/or analyzed during the current study are available from the corresponding author on reasonable request.
